# Supplementary material for: Acquisition, co-option, and duplication of the rtx toxin system and the emergence of virulence in Kingella
Source: Nat Commun. 2023 Jul 17;14:4281. doi: 10.1038/s41467-023-39939-8 (PMC10352306; doi:10.1038/s41467-023-39939-8)
Supplement: Supplementary file 1 — Supplementary Information [file 41467_2023_39939_MOESM1_ESM.pdf]

Supplemental Figures

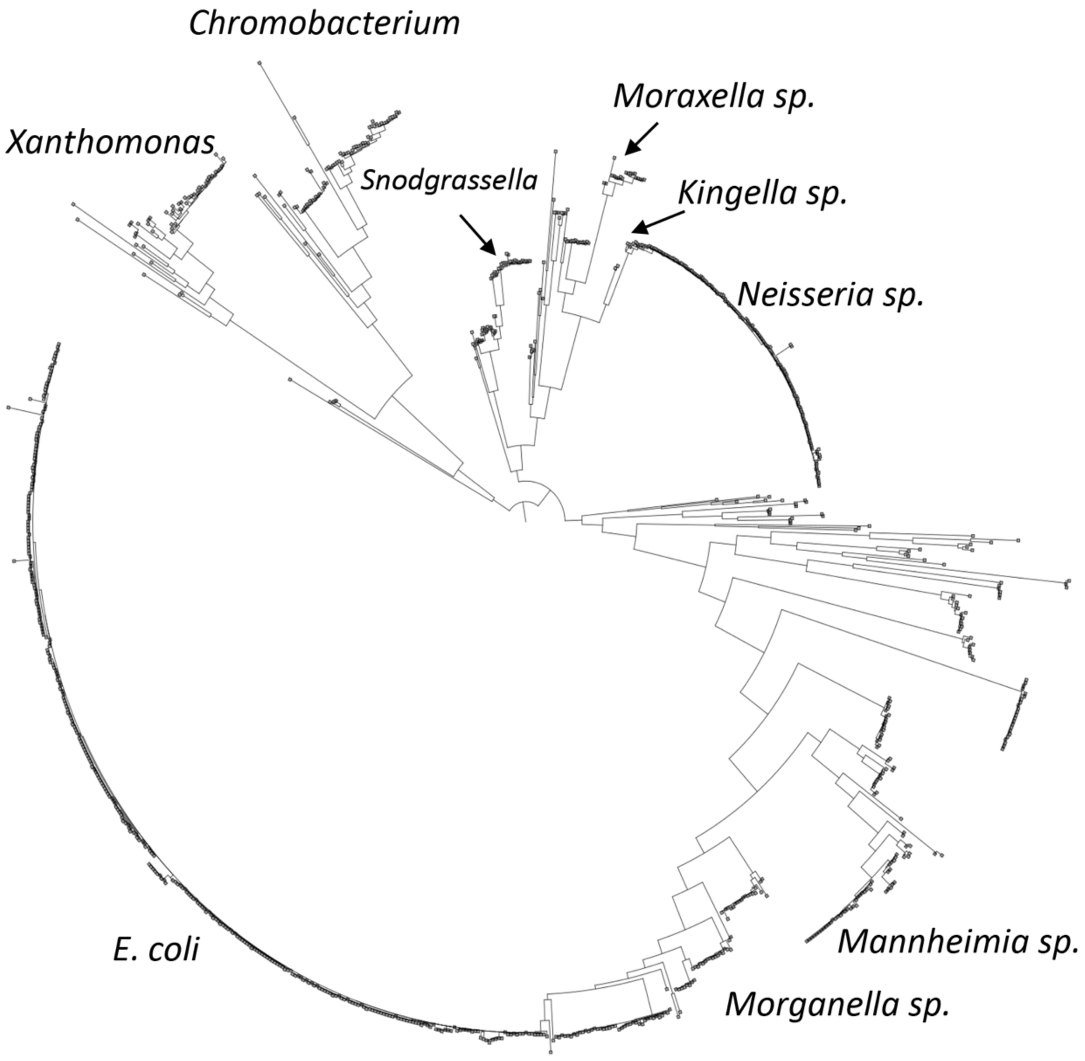

Fig. S1. RTX-associated genes show distinct homologs from diverse bacterial species. BlastP was used to identify homologs of RtxB in diverse bacterial species. Homologous sequences were downloaded and used for phylogenetic reconstruction allowing us to identify RTX proteins across at least 134 diverse bacterial genera with IQTRee. Taxonomic classifications of interest are annotated for each phylogeny. The tree is rooted by *E. coli* isolateswith bootstrap values masked for clarity. Newick formatted tree is available in the source data files.

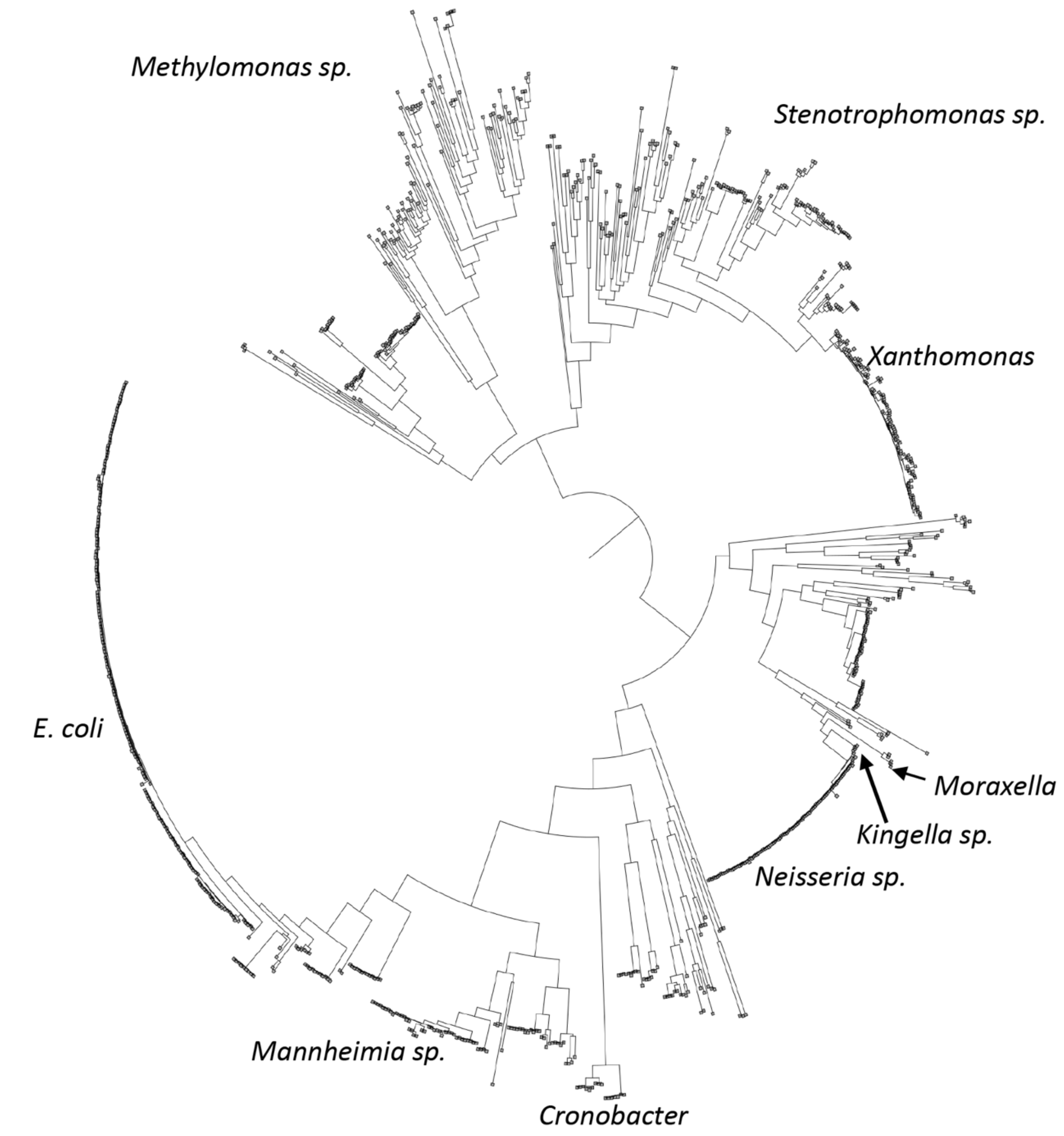

Fig. S2. RTX-associated genes show distinct homologs from diverse bacterial species. BlastP was used to identify homologs of RtxD in diverse bacterial species. Homologous sequences were downloaded and used for phylogenetic reconstruction allowing us to identify RTX proteins across at least 134 diverse bacterial genera with IQTRee. Taxonomic classifications of interest are annotated for each phylogeny. The tree is rooted by *E. coli* and *Vibrio* species*,* with bootstrap values masked for clarity. Newick formatted tree is available in the source data files.

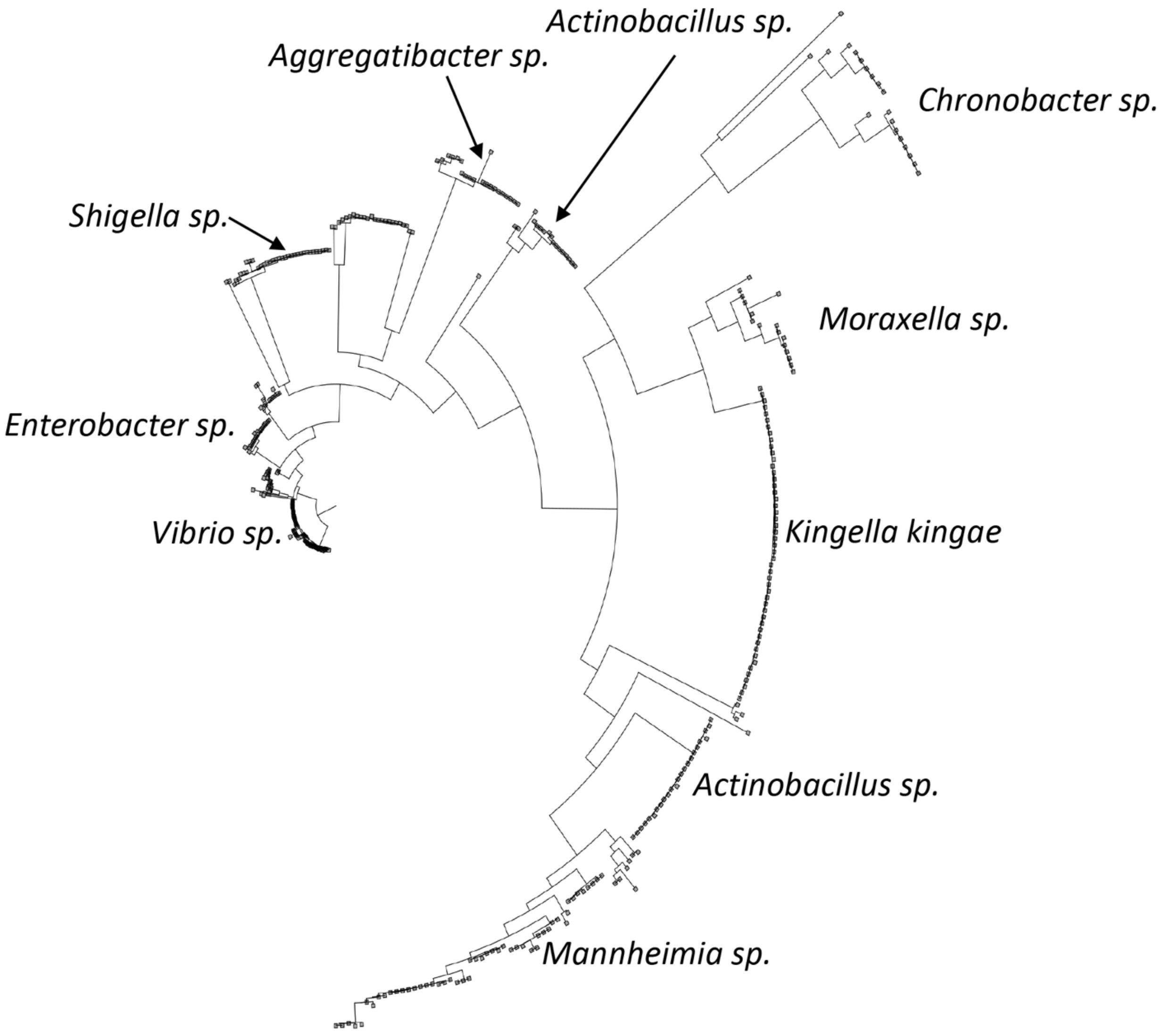

Fig. S3. RTX-associated genes show distinct homologs from diverse bacterial species. BlastP was used to identify homologs of RtxA in diverse bacterial species. Homologous sequences were downloaded and used for phylogenetic reconstruction allowing us to identify RTX proteins across at least 134 diverse bacterial genera with IQTRee. Taxonomic classifications of interest are annotated for each phylogeny. The tree is rooted by *Vibrio* species, with bootstrap values masked for clarity. Newick formatted tree is available in the source data files.

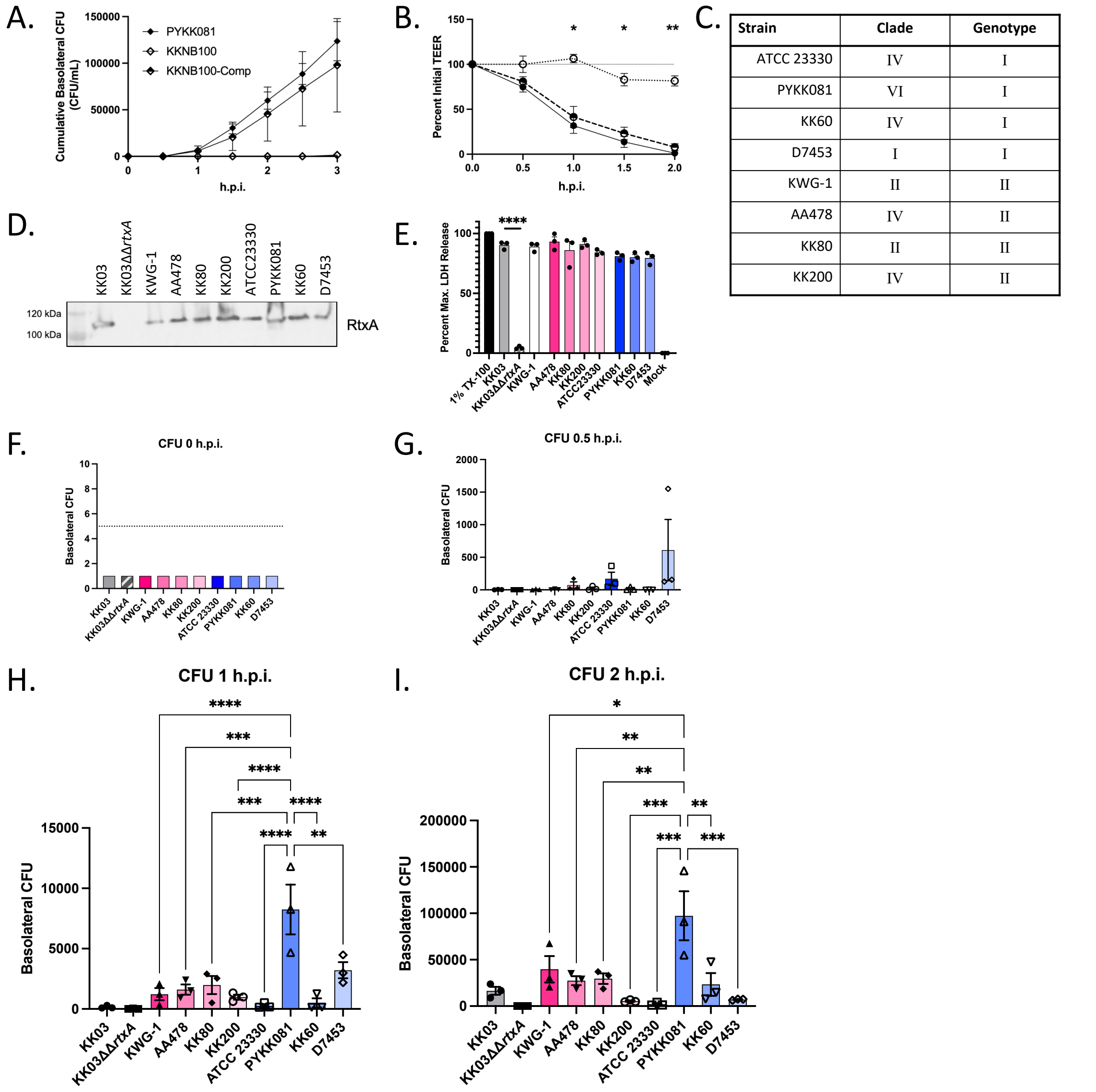

Strain	Clade	Genotype
ATCC 23330	IV	I
PYKK081	VI	I
KK60	IV	I
D7453	I	I
KWG-1	II	II
AA478	IV	II
KK80	II	II
KK200	IV	II

Fig. S4. *In vitro* characterizations of RTX copy number variability. A-B. 16HBE14o- cells were cultured at an ALI and infected at a multiplicity of infection (MOI) of ~10 on the apical surface in 1x MEM with PYKK081, KKNB100 (Δ*rtxA*), or KKNB100-Comp. Over the course of infection, cumulative transited CFUs in the basolateral chamber (A) was monitored every 30 minutes for three hours post infection (h.p.i). Transepithelial resistance (B) was monitored every 30 mins for 2 h.p.i. PYKK081 WT is shown in filled diamonds, KKNB100 (Δ*rtxA*) is shown in open filled diamonds, and KKNB100-Comp is shown in half filled diamonds. **p*=0.01, ***p*=0.004. C. A clinical panel of isolates was constructed to include four diverse isolates from genotypes I and II, respectively. D. RtxA levels secreted into the culture supernatant were determined via Western blot. E. LDH release by 16HBE-14o- cells was determined using the panel of clinical isolates. Monolayers were infected for 1 hour at an MOI of ~40. LDH was quantified. Statistics calculated with a one-way ANOVA, ****$p<0.0001$. F-I. Bacterial transit across an epithelial barrier was monitored over the course of infection by the genotype I and genotype II clinical isolates characterized above by enumeration of basolateral CFUs. CFUs were calculated 0 m.p.i. (F), 30 m.p.i. (G), 1 h.p.i. (H), and 2 h.p.i. (I). Statistics calculated with a one-way ANOVA. For (H) **p=0.005, ***p=0.0004,

****$p<0.0001$. For (I) *$p=0.03$, **$p=0.006$,***$p=0.0004$. All *in vitro* experiments include at least 3, independent biological replicates; averages are plotted with error bars representing the standard error of the mean (±1 SEM). Source data are provided in the attached source data file.
