# Supplementary material for: Acquisition, co-option, and duplication of the rtx toxin system and the emergence of virulence in Kingella
Source: Nat Commun. 2023 Jul 17;14:4281. doi: 10.1038/s41467-023-39939-8 (PMC10352306; doi:10.1038/s41467-023-39939-8)
Supplement: Supplementary file 3 — Description of Additional Supplementary Files [file 41467_2023_39939_MOESM3_ESM.pdf]

Description of Additional Supplementary Files:

Supplementary Data 1: Strains used in this study.

Supplementary Data 2: Assembly statistics calculated by Quast for strains used in this study. Assembly statistics were calculated in reference to *K. kingae* KWG-1 (LN869922.1)

Supplementary Data 3: International isolates genotyped in this study.

Supplementary Data 4: Pairwise protein similarities were calculated with Needle for representative sequences of genes of interest.

Supplementary Data 5: Roary output for Accessory genes (present in >117 strains).

Supplementary Data 6: Roary output for Accessory genes (present in <117 strains).

Supplementary Data 7: Gene Enrichment By Clade. Scoary was used to determine diagnostic genes for each clade. Bonferroni-corrected p-values are shown. p>0.001 were considered statistically significant.
