# Supplementary material for: Acquisition, co-option, and duplication of the rtx toxin system and the emergence of virulence in Kingella
Source: Nat Commun. 2023 Jul 17;14:4281. doi: 10.1038/s41467-023-39939-8 (PMC10352306; doi:10.1038/s41467-023-39939-8)
Supplement: Supplementary file 5 — Reporting Summary [file 41467_2023_39939_MOESM5_ESM.pdf]

nature portfolio

Corresponding author(s): Paul J. Planet, M.D., Ph.D.
Last updated by author(s): 2023-06-06

Reporting Summary

Nature Portfolio wishes to improve the reproducibility of the work that we publish. This form provides structure for consistency and transparency in reporting. For further information on Nature Portfolio policies, see our Editorial Policies and the Editorial Policy Checklist.

Statistics

For all statistical analyses, confirm that the following items are present in the figure legend, table legend, main text, or Methods section.

n/a	Confirmed	
☐	☒	The exact sample size (*n*) for each experimental group/condition, given as a discrete number and unit of measurement
☐	☒	A statement on whether measurements were taken from distinct samples or whether the same sample was measured repeatedly
☐	☒	The statistical test(s) used AND whether they are one- or two-sided<br>*Only common tests should be described solely by name; describe more complex techniques in the Methods section.*
☒	☐	A description of all covariates tested
☐	☒	A description of any assumptions or corrections, such as tests of normality and adjustment for multiple comparisons
☐	☒	A full description of the statistical parameters including central tendency (e.g. means) or other basic estimates (e.g. regression coefficient) AND variation (e.g. standard deviation) or associated estimates of uncertainty (e.g. confidence intervals)
☐	☒	For null hypothesis testing, the test statistic (e.g. *F*, *t*, *r*) with confidence intervals, effect sizes, degrees of freedom and *P* value noted<br>*Give P values as exact values whenever suitable.*
☒	☐	For Bayesian analysis, information on the choice of priors and Markov chain Monte Carlo settings
☒	☐	For hierarchical and complex designs, identification of the appropriate level for tests and full reporting of outcomes
☒	☐	Estimates of effect sizes (e.g. Cohen's *d*, Pearson's *r*), indicating how they were calculated

Our web collection on statistics for biologists contains articles on many of the points above.

Software and code

Policy information about availability of computer code

Data collection	Publicly available gene and proteins sequences analyzed in this study were collected using the online BLAST suite hosted by NCBI.
Data analysis	Sequenceing data was analyzed with Trim Galore v.0.6.4, SPAdes v.3.14, Prokka v. 1.14.6, FastANI v.1.32, R v.4.1.2, Quast v.5.0.2, Roary v.3.13.0, Scoary v.1.6.16, Snippy v.4.6.0, RAxML v.8.2.4, MAFFT v.7, and MinKNOW v22.03.6. Statistics were calcualted in GraphPad Prism v.9.2.0 or R suite.

For manuscripts utilizing custom algorithms or software that are central to the research but not yet described in published literature, software must be made available to editors and reviewers. We strongly encourage code deposition in a community repository (e.g. GitHub). See the Nature Portfolio guidelines for submitting code & software for further information.

Data

Policy information about availability of data

All manuscripts must include a data availability statement. This statement should provide the following information, where applicable:

- Accession codes, unique identifiers, or web links for publicly available datasets
- A description of any restrictions on data availability
- For clinical datasets or third party data, please ensure that the statement adheres to our policy

Files containing phylogenetic trees in Newick format and sequence alignments are available at Github (https://github.com/danmorreale/Kingella-Phylogenomics). All sequencing data generated in this study is publicly available on the National Center for Biotechnology Information's (NCBI) Sequence Read Archive (SRA) under Bioproject PRJNA896475. All strains presented in this study are available from the authors by request. The VFDB and MEGARes database are distributed as part of Abricate.

Human research participants

Policy information about studies involving human research participants and Sex and Gender in Research.

Reporting on sex and gender	Not Applicable.
Population characteristics	Not Applicable.
Recruitment	Not Applicable.
Ethics oversight	Not Applicable.

Note that full information on the approval of the study protocol must also be provided in the manuscript.

Field-specific reporting

Please select the one below that is the best fit for your research. If you are not sure, read the appropriate sections before making your selection.

☒ Life sciences ☐ Behavioural & social sciences ☐ Ecological, evolutionary & environmental sciences

For a reference copy of the document with all sections, see nature.com/documents/nr-reporting-summary-flat.pdf

Life sciences study design

All studies must disclose on these points even when the disclosure is negative.

Sample size	Sample size for all experiments was based on previously published literature. Please see Chang et al. 2014 (doi: 10.1128/IAI.01636-14) and Munoz et al. 2018 (doi: 10.1128/IAI.00100-18).
Data exclusions	Whole genome sequencing reads were excluded from analysis if, after Illumina sequencing adapters were trimmed, the PHRED quality score of the read was less than 15. Four genomes were excluded as the final assembled genome size was < 1 Mb (~50% of average *K. kingae* genome size).
Replication	All experiments were performed in at least three, independent, biological replicates. All replicate experiments were successful. In house sequencing of genomes already publicly available serve as additional replicates for phylogenetic reconstruction.
Randomization	In vitro experiments using cultured 16HBE-14o- cell were randomly shuffled prior to infection to reduce variation during seeding and growth. For all other experiments, samples were infected in a different, random order for each replicate to reduce variation in infection time.
Blinding	Investigators were not blinded for any of the experiments performed in this work. Strains were selected for experimental characterization based on RtxA copy-number variation, eliminating the possibility of blinding all investigators to this data.

Behavioural & social sciences study design

All studies must disclose on these points even when the disclosure is negative.

Study description	Not applicable.
Research sample	Not applicable.
Sampling strategy	Not applicable.

Data collection	Not applicable.
Timing	Not applicable.
Data exclusions	Not applicable.
Non-participation	Not applicable.
Randomization	Not applicable.

Ecological, evolutionary & environmental sciences study design

All studies must disclose on these points even when the disclosure is negative.

Study description	This study examines the events that lead to the evolution of pathogenesis by *Kingella* species, with particular focus on the the horizontal gene transfer events that result in hemolysis by *K. kingae.*
Research sample	Samples were selected from the strain collection of *Kingella* isolates housed in the St. Geme lab at the Children's Hospital of Philadelphia, which includes global isolates collected over the previous 30+ years from clinical isolates recovered globally. Isolates were selected to include an approximately equal mixture of invasive and carriage isolates, and to cover the largest span of time possible.
Sampling strategy	All bacterial strains used in this study were sampled from a collection of clinical *Kingella* isolates housed in the St. Geme Lab at the Children's Hospital of Philadelphia.
Data collection	Sequencing data was collected collected from gDNA prepared by B.K. Where available, metadata was collected from prior publications.
Timing and spatial scale	Not applicable.
Data exclusions	Sequence read data was excluded from analysis if the PHRED score fell below 15, a cutoff established by others in the literature.
Reproducibility	To ensure reproducibility in sequencing data, isolates were selected from the collection which had already been sequenced by other groups and were publicly available on NCBI (denoted by "_N"). Sequenced isolates from our collection were compared to public data.
Randomization	Not applicable.
Blinding	No blinding was used in these studies.

Did the study involve field work? ☐ Yes ☒ No

Field work, collection and transport

Field conditions	Not applicable.
Location	Not applicable.
Access & import/export	Not applicable.
Disturbance	Not applicable.

Reporting for specific materials, systems and methods

We require information from authors about some types of materials, experimental systems and methods used in many studies. Here, indicate whether each material, system or method listed is relevant to your study. If you are not sure if a list item applies to your research, read the appropriate section before selecting a response.

Materials & experimental systems

n/a	Involved in the study
☐	☒ Antibodies
☐	☒ Eukaryotic cell lines
☒	☐ Palaeontology and archaeology
☐	☒ Animals and other organisms
☒	☐ Clinical data
☒	☐ Dual use research of concern

Methods

n/a	Involved in the study
☒	☐ ChIP-seq
☒	☐ Flow cytometry
☒	☐ MRI-based neuroimaging

Antibodies

Antibodies used	GP-23, a custom guinea pig antibody generated by Cocalico Biologicals, Inc., and HRP conjugated to Goat-αGP (Sigma A7289) .
Validation	GP-23 was validated by western blot and ELISA against purified, recombinant RtxA, as well as against RtxA-deficient mutant strains. αgp-HRP was purchased from Sigma and was validated by the manufacturer.

Eukaryotic cell lines

Policy information about cell lines and Sex and Gender in Research

Cell line source(s)	16HBE-14o- cells were used from an in-house collection of cell lines. Cells are human, bronchial epithelial in origin and are commercially available from Sigma-Aldrich (SCC150).
Authentication	16HBE-14o- cells were authenticated by the American Tissue Culture Collection by human STR analysis.
Mycoplasma contamination	Cell stocks tested negative for mycoplasma contamination.
Commonly misidentified lines (See ICLAC register)	None.

Palaeontology and Archaeology

Specimen provenance	Not applicable.
Specimen deposition	Not applicable.
Dating methods	Not applicable.

☐ Tick this box to confirm that the raw and calibrated dates are available in the paper or in Supplementary Information.

Ethics oversight	Not applicable.

Note that full information on the approval of the study protocol must also be provided in the manuscript.

Animals and other research organisms

Policy information about studies involving animals; ARRIVE guidelines recommended for reporting animal research, and Sex and Gender in Research

Laboratory animals	5-day-old Sprague-dawley rats were purchased from Charles River Labs, and were co-housed with their dams.

Wild animals	No wild animals were used in this study.
Reporting on sex	Rat pups were not segregated by sex in this study, following the protocol used in prior studies that employed this model. There is no known effect on morbidity or mortality by *K. kingae* during invasive disease based on animal sex.
Field-collected samples	No field-collected samples were used in this study.
Ethics oversight	Procedures were approved by the Children's Hospital of Philadelphia Institutional Animal Care and Use Committee under protocol IAC 19-001050.

Note that full information on the approval of the study protocol must also be provided in the manuscript.

Clinical data

Policy information about clinical studies

All manuscripts should comply with the ICMJE guidelines for publication of clinical research and a completed CONSORT checklist must be included with all submissions.

Clinical trial registration	Not applicable.
Study protocol	Not applicable.
Data collection	Not applicable.
Outcomes	Not applicable.

Dual use research of concern

Policy information about dual use research of concern

Hazards

Could the accidental, deliberate or reckless misuse of agents or technologies generated in the work, or the application of information presented in the manuscript, pose a threat to:

No	Yes	
☒	☐	Public health
☒	☐	National security
☒	☐	Crops and/or livestock
☒	☐	Ecosystems
☒	☐	Any other significant area

Experiments of concern

Does the work involve any of these experiments of concern:

No	Yes	
☒	☐	Demonstrate how to render a vaccine ineffective
☒	☐	Confer resistance to therapeutically useful antibiotics or antiviral agents
☒	☐	Enhance the virulence of a pathogen or render a nonpathogen virulent
☒	☐	Increase transmissibility of a pathogen
☒	☐	Alter the host range of a pathogen
☒	☐	Enable evasion of diagnostic/detection modalities
☒	☐	Enable the weaponization of a biological agent or toxin
☒	☐	Any other potentially harmful combination of experiments and agents

ChIP-seq

Data deposition

☐ Confirm that both raw and final processed data have been deposited in a public database such as GEO.

☐ Confirm that you have deposited or provided access to graph files (e.g. BED files) for the called peaks.

Data access links<br>*May remain private before publication.*	*For "Initial submission" or "Revised version" documents, provide reviewer access links. For your "Final submission" document, provide a link to the deposited data.*
Files in database submission	*Provide a list of all files available in the database submission.*
Genome browser session<br>(e.g. UCSC)	*Provide a link to an anonymized genome browser session for "Initial submission" and "Revised version" documents only, to enable peer review. Write "no longer applicable" for "Final submission" documents.*

Methodology

Replicates	*Describe the experimental replicates, specifying number, type and replicate agreement.*
Sequencing depth	*Describe the sequencing depth for each experiment, providing the total number of reads, uniquely mapped reads, length of reads and whether they were paired- or single-end.*
Antibodies	*Describe the antibodies used for the ChIP-seq experiments; as applicable, provide supplier name, catalog number, clone name, and lot number.*
Peak calling parameters	*Specify the command line program and parameters used for read mapping and peak calling, including the ChIP, control and index files used.*
Data quality	*Describe the methods used to ensure data quality in full detail, including how many peaks are at FDR 5% and above 5-fold enrichment.*
Software	*Describe the software used to collect and analyze the ChIP-seq data. For custom code that has been deposited into a community repository, provide accession details.*

Flow Cytometry

Plots

Confirm that:

☐ The axis labels state the marker and fluorochrome used (e.g. CD4-FITC).

☐ The axis scales are clearly visible. Include numbers along axes only for bottom left plot of group (a 'group' is an analysis of identical markers).

☐ All plots are contour plots with outliers or pseudocolor plots.

☐ A numerical value for number of cells or percentage (with statistics) is provided.

Methodology

Sample preparation	*Describe the sample preparation, detailing the biological source of the cells and any tissue processing steps used.*
Instrument	*Identify the instrument used for data collection, specifying make and model number.*
Software	*Describe the software used to collect and analyze the flow cytometry data. For custom code that has been deposited into a community repository, provide accession details.*
Cell population abundance	*Describe the abundance of the relevant cell populations within post-sort fractions, providing details on the purity of the samples and how it was determined.*
Gating strategy	*Describe the gating strategy used for all relevant experiments, specifying the preliminary FSC/SSC gates of the starting cell population, indicating where boundaries between "positive" and "negative" staining cell populations are defined.*

☐ Tick this box to confirm that a figure exemplifying the gating strategy is provided in the Supplementary Information.

Magnetic resonance imaging

Experimental design

Design type	*Indicate task or resting state; event-related or block design.*

Design specifications | *Specify the number of blocks, trials or experimental units per session and/or subject, and specify the length of each trial or block (if trials are blocked) and interval between trials.*

Behavioral performance measures | *State number and/or type of variables recorded (e.g. correct button press, response time) and what statistics were used to establish that the subjects were performing the task as expected (e.g. mean, range, and/or standard deviation across subjects).*

Acquisition

Imaging type(s) | *Specify: functional, structural, diffusion, perfusion.*

Field strength | *Specify in Tesla*

Sequence & imaging parameters | *Specify the pulse sequence type (gradient echo, spin echo, etc.), imaging type (EPI, spiral, etc.), field of view, matrix size, slice thickness, orientation and TE/TR/flip angle.*

Area of acquisition | *State whether a whole brain scan was used OR define the area of acquisition, describing how the region was determined.*

Diffusion MRI ☐ Used ☐ Not used

Preprocessing

Preprocessing software | *Provide detail on software version and revision number and on specific parameters (model/functions, brain extraction, segmentation, smoothing kernel size, etc.).*

Normalization | *If data were normalized/standardized, describe the approach(es): specify linear or non-linear and define image types used for transformation OR indicate that data were not normalized and explain rationale for lack of normalization.*

Normalization template | *Describe the template used for normalization/transformation, specifying subject space or group standardized space (e.g. original Talairach, MNI305, ICBM152) OR indicate that the data were not normalized.*

Noise and artifact removal | *Describe your procedure(s) for artifact and structured noise removal, specifying motion parameters, tissue signals and physiological signals (heart rate, respiration).*

Volume censoring | *Define your software and/or method and criteria for volume censoring, and state the extent of such censoring.*

Statistical modeling & inference

Model type and settings | *Specify type (mass univariate, multivariate, RSA, predictive, etc.) and describe essential details of the model at the first and second levels (e.g. fixed, random or mixed effects; drift or auto-correlation).*

Effect(s) tested | *Define precise effect in terms of the task or stimulus conditions instead of psychological concepts and indicate whether ANOVA or factorial designs were used.*

Specify type of analysis: ☐ Whole brain ☐ ROI-based ☐ Both

Statistic type for inference (See Eklund et al. 2016) | *Specify voxel-wise or cluster-wise and report all relevant parameters for cluster-wise methods.*

Correction | *Describe the type of correction and how it is obtained for multiple comparisons (e.g. FWE, FDR, permutation or Monte Carlo).*

Models & analysis

n/a	Involved in the study
☐	☐ Functional and/or effective connectivity
☐	☐ Graph analysis
☐	☐ Multivariate modeling or predictive analysis

Functional and/or effective connectivity | *Report the measures of dependence used and the model details (e.g. Pearson correlation, partial correlation, mutual information).*

Graph analysis | *Report the dependent variable and connectivity measure, specifying weighted graph or binarized graph, subject- or group-level, and the global and/or node summaries used (e.g. clustering coefficient, efficiency, etc.).*

Multivariate modeling and predictive analysis | *Specify independent variables, features extraction and dimension reduction, model, training and evaluation metrics.*
