# Supplementary figures and images for: Acquisition, co-option, and duplication of the rtx toxin system and the emergence of virulence in Kingella
Source: Nat Commun. 2023 Jul 17;14:4281. doi: 10.1038/s41467-023-39939-8 (PMC10352306; doi:10.1038/s41467-023-39939-8)

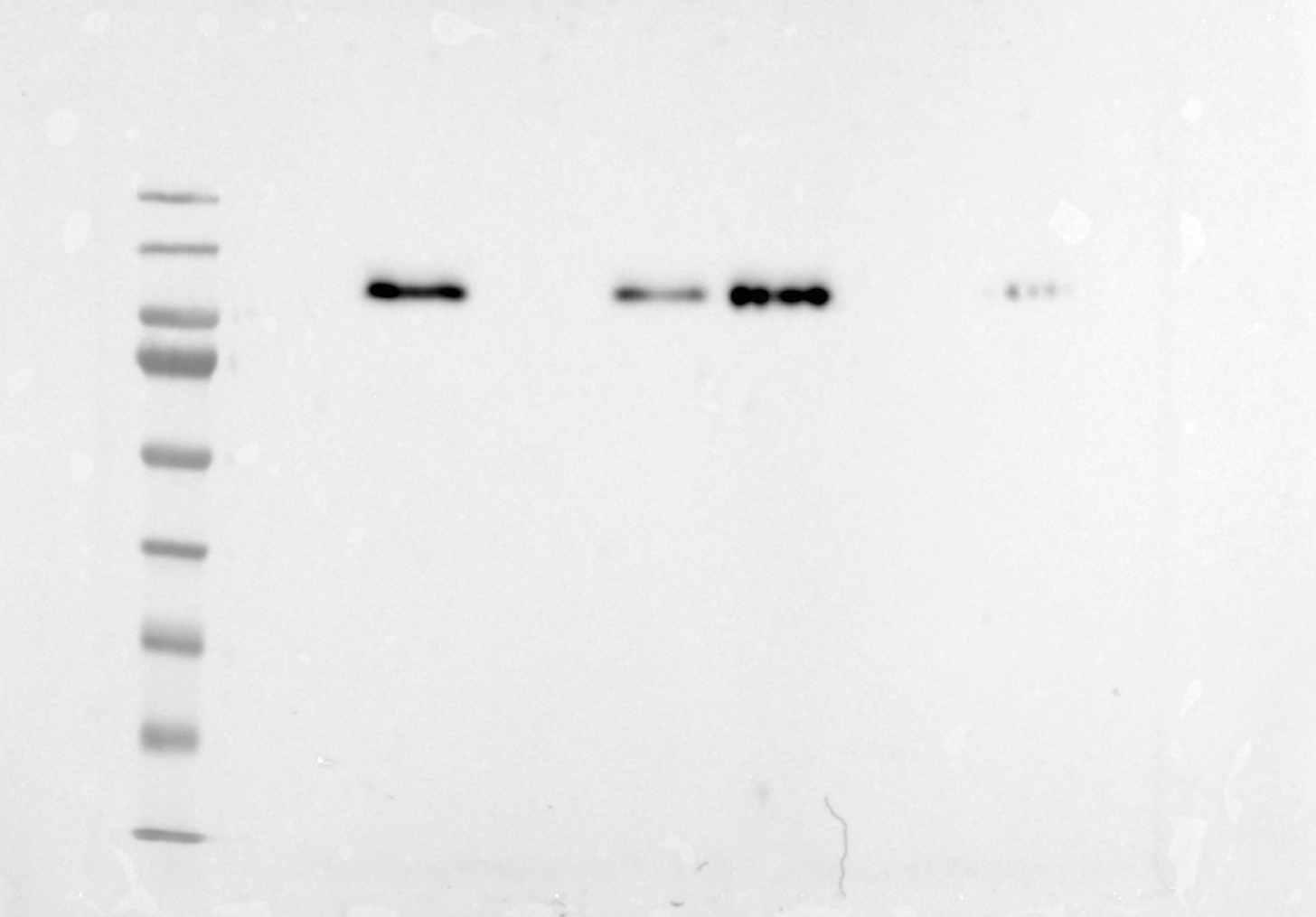

Supplement: Supplementary file 6 — Source Data [file 41467_2023_39939_MOESM6_ESM.zip › Source Data/Figure4a.tif]

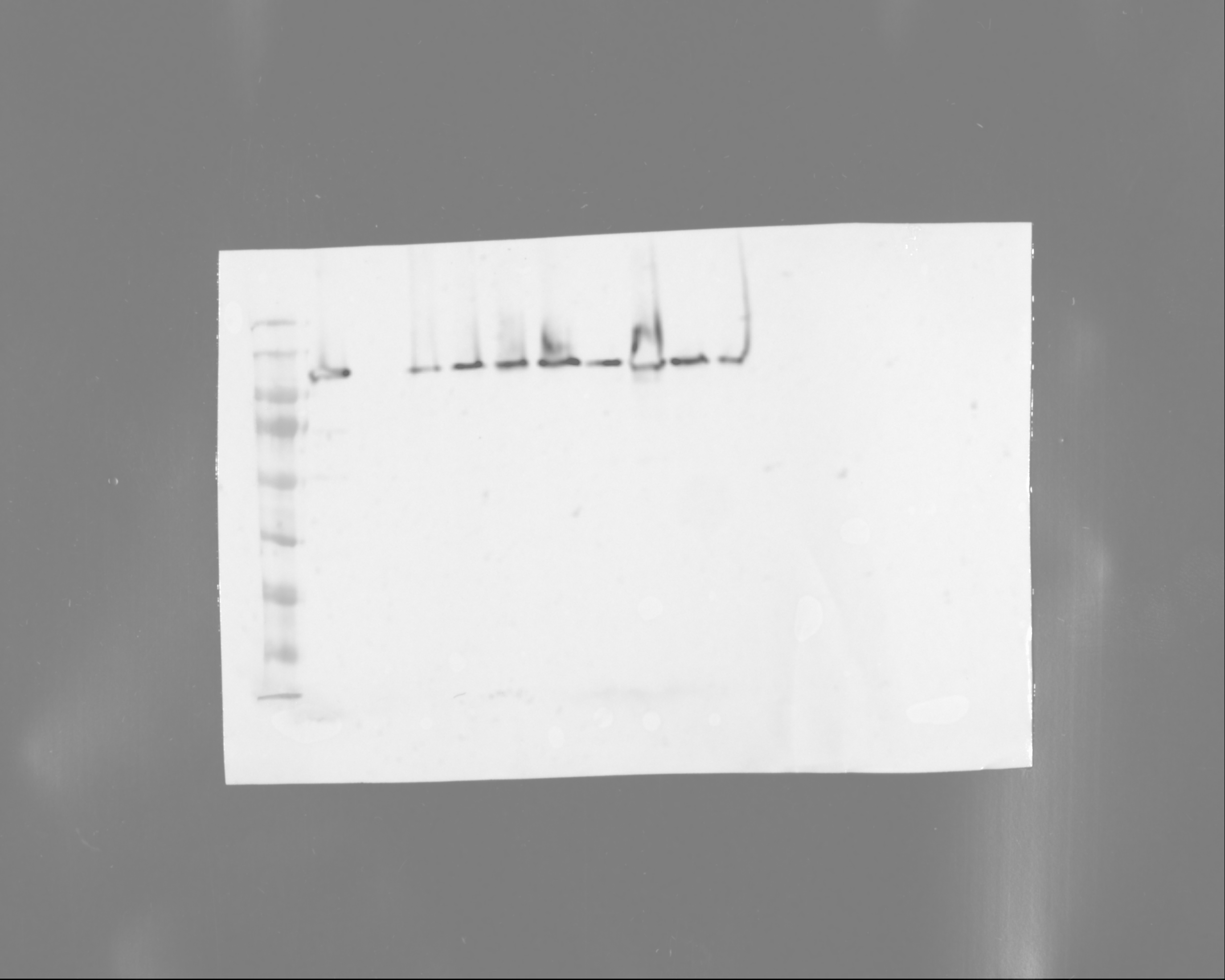

Supplement: Supplementary file 6 — Source Data [file 41467_2023_39939_MOESM6_ESM.zip › Source Data/FigureS4a.tif]
